# Therapeutic efficacy of rose oil: A comprehensive review of clinical evidence

**Published:** 2017

**Authors:** Safieh Mohebitabar, Mahboobeh Shirazi, Sodabeh Bioos, Roja Rahimi, Farhad Malekshahi, Fatemeh Nejatbakhsh

**Affiliations:** 1 *Department of Iranian Traditional Medicine, School of Traditional Medicine, Tehran University of Medical Sciences, Tehran, Iran*; 2 *Maternal, Fetal, and Neonatal Research Center, Tehran University of Medical Sciences, Tehran, Iran*; 3 *Department of Traditional Pharmacy, School of Traditional Medicine, Tehran University of Medical Sciences, Tehran, Iran*; 4 *Department of management, School of management and accounting, Emam Hosein University, Tehran, Iran*

**Keywords:** Rosa damascene, Rose oil, Comprehensive review, Human studies, Clinical trial

## Abstract

**Objective:**

Rose oil is obtained from the petals of difference Rosa species especially *Rosa centifolia *L. and *Rosa damascena *Mill. Various pharmacological properties have been attributed to rose oil. The aim of the present study was to review the rose oil therapeutic effects which had been clinically evaluated in trial studies.

**Materials and Methods:**

Google scholar, PubMed, Cochrane Library, and Scopus were searched for human studies which have evaluated the therapeutic effects of rose oil and published in English language until August 2015.

**Results:**

Thirteen clinical trials (772 participants) were included in this review. Rose oil was administered via inhalation or used topically. Most of the studies (five trials) evaluated the analgesic effect of rose oil. Five studies evaluated the physiological relaxation effect of rose oil. Anti-depressant, psychological relaxation, improving sexual dysfunction, and anti-anxiety effects were the other clinical properties reported for rose oil.

**Conclusion:**

Numerous studies on the pharmacological properties of rose oil have been done in animals, but studies in humans are few. In this study, it was observed that rose oil had physiological and psychological relaxation, analgesic and anti-anxiety effects. To obtain conclusive results on the efficacy and safety of rose oil, further clinical trials with larger sample size and better designation are required.

## Introduction

Rosa species commonly known as rose (Family Rosaceae) are among the most popular and widely used medicinal plants all over the world. They are originated from the Middle East but are cultivated all over the world (Krussman, 1981 ▶). Rose oil is the essential oil extracted from the petals of Rosa species especially *R. damascena* and *R. centifolia*. Some historical evidence shows that rose oil is originated from Greece (Zargari, 1992 ▶). Currently, the main producing countries of this essential oil are Bulgaria, Turkey, and Morocco. This oil is semisolid, pale, yellow, and very expensive (Baydar, 2005 ▶).

The most important components of rose oil are terpenes, glycosides, flavonoids, and anthocyanins (Almasirad et al., 2007 ▶; Knapp et al., 1998 ▶; Schiber et al., 2005 ▶). In a study carried out on the essential oil of *R. damascena* in Kashan region of Iran, 95 components were reported and the most abundant ones were β-citronellol (14.5-47.5%), nonadecane (10.5-40.5%), geraniol (5.5-18%) (Loghmani-Khouzaniet al., 2007 ▶). 

In Persian Medicine , rose oil has been alleged to have anti-inflammatory, anti-infective and wound healing activities and has been used for relieving headache, hemorrhoids, inflammatory conditions of gastrointestinal tract, and muscular pain (Agili Shirazi, 2008 ▶; Ibn Sina, 2005 ▶). 

Pharmacological activities of rose oil have been evaluated by several *in vitro* and *in vivo* studies (Maleev et al., 1972 ▶; Boskabady et al., 2006 ▶). Some studies have demonstrated its effects on the central nervous system (CNS) including hypnotic, anti-convulsant, anti-depressant, anti-anxiety, analgesic activities as well as alleviation of morphine withdrawal signs (Abbasi Maleki et al., 2013 ▶; De Almeida et al., 2004 ▶; Naziroglu et al., 2013 ▶; Ramezani et al., 2008 ▶; Umezu et al., 2002 ▶ ; Boskabady et al., 2011 ▶). Rose oil has revealed wide spectrum of antibacterial and antifungal properties against some pathogens including *Bacillus cereus*, *Pseudomonas aeruginosa*, *P. fluorescens*, *Penicillium notatum*, *Aspergillus niger* and *Candida albicans* (Eris and Ulusoy 2013 ▶; Gochev et al., 2008 ▶; Shohayeb et al., 2014 ▶; Ulusoy et al., 2009 ▶; Uniyal et al., 2013 ▶; Zu et al., 2010 ▶). Rose oil also enhanced ileum contractions and gastrointestinal motility in rats (Sadraei et al., 2013 ▶). Inhalation of rose oil showed protective effects against damages caused by exposure to formaldehyde in male reproductive system (Köse et al., 2012 ▶). 

The aim of the present study is to comprehensively review the effects of rose oil in human studies. 

## Methods

**Figure 1:**
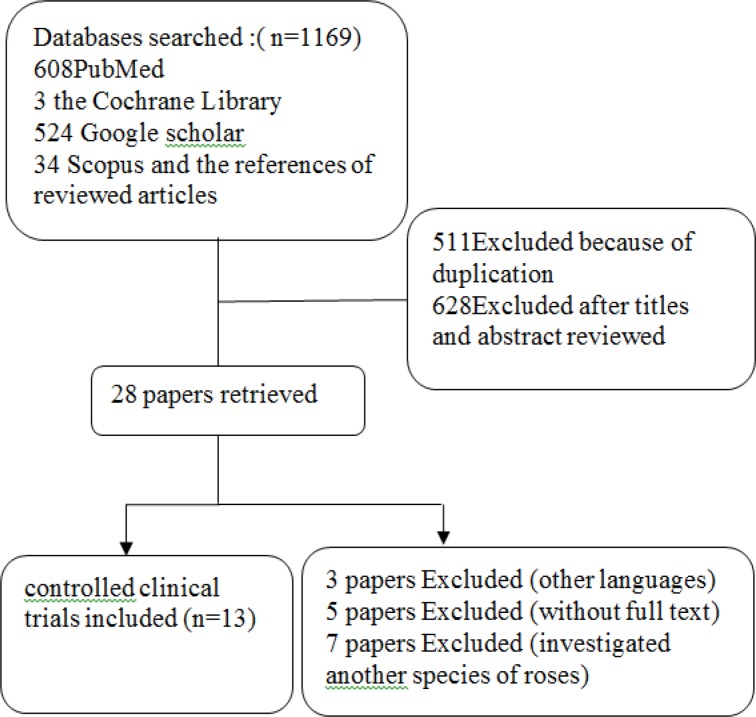
The structure of the literature review

A literature review was performed until August 2015. We searched Pubmed, the Cochrane Library, Google scholar, and Scopus for studies evaluating the effects of rose oil in human subjects. Only, The the papers written in English were considered. The reference list from retrieved articles and review articles has been also searched. We did a Boolean search using the term “or” / “and” to explore (search by subject heading) and map (search by keyword) the MeSH headings. The search terms were: “rosa” or “rose” or “rose oil” and “volatile oil” or "essential oil" and "clinical" or "human". This search excluded case reports, comments, editorials, and letters using the Boolean operator “not” (Figure 1). 

## Results

The electronic search yielded 28 items. Papers without full text, articles that were not written in English and articles that had been investigated other species of Rosa were excluded. Thirteen clinical trials (772 participants) were included. The included studies evaluated rose oil via different approaches. Rose oil has been administered as aromatherapy or topically in these studies. It has been demonstrated that essential oils can be absorbed into the body via the skin or the olfactory system (Dye,1997 ▶; Tisserand,1996 ▶). Many studies found that olfactory stimulation by essential oils could produce instant changes in physiological parameters including muscle tension, blood pressure (BP), pulse rate, skin temperature, skin blood flow, electrodermal activity, and brain activity (Diego et al., 1998 ▶; Field et al., 2005 ▶; Lorig and Schwartz, 1988 ▶; Tisserand, 1996 ▶; Van Toller et al., 1993 ▶). Table 1 shows a summary of these studies.

**Studies focusing on anti-depressant effect**

Farnia et al. (2015a) ▶ showed that *R. damascena* oil improves the symptoms of depression and selective serotonin reuptake inhibitors-induced sexual dysfunction (SSRI-ISD) in 60 male patients who were suffering from major depressive disorder (MDD) and were being treated with SSRIs. In another similar study on 50 female patients suffered from depression and SSRI-ISD, sexual desire, sexual orgasms and sexual satisfaction increased, and pain decreased by rose oil inhalation. The effect of rose oil in improvement of sexual function in male patients was more than that in female patients (Farnia et al., 2015b ▶). Some mechanisms have been also suggested for anti-depressant activity of rose oil including antagonistic effect on the stimulation of the post-synaptic 5-HT2 and 5-HT3 receptors as well as antagonistic effect on the cortico-limbic 5-HT receptors, which may also affect sexual behavior and could be responsible for increasing sexual desire, ejaculation, and orgasm. Moreover, rose oil increased the release of dopamine and norepinephrine in the substantia nigra, and inhibited nitric oxide synthase (Farnia et al., 2015b ▶)

**Studies focusing on analgesic effect**

In addition to conventional therapy, inhalation of the fragrance of rose essential oil by eighty patients with renal colic in the emergency room, effectively reduced renal colic pain (Ayan et al., 2013 ▶).

It was found that massage with rose oil reduces the severity of primary dysmenorrhea compared to massage therapy alone in 75 students (Sadeghi AvalShahr et al., 2015 ▶). Rose oil in combination with other essential oils also showed beneficial effects in reducing menstrual pain and bleeding (Marzouk et al., 2013 ▶; Kim et al., 2011 ▶).

A double-blind placebo controlled clinical trial compared the effects of rose oil inhalation with inhalation of almond oil. The results demonstrated reduction in postoperative pain in 32 3-6-year-old children without any significant side effects (Marofi et al., 2015 ▶). The possible mechanisms for reducing pain by rose oil inhalation are stimulating the olfactory system, increasing parasympathetic activity, releasing neurotransmitters such as enkephalin and endorphin as well as reducing sympathetic activity and the release of cortisol and noradrenalin (Ikei et al., 2014 ▶; Lee et al., 2011 ▶; Park et al., 2007 ▶; Tsunetsugu et al., 2007 ▶).

**Table1:** Therapeutic effect of *Rosa damascena* oil in human stadies

**Author** **(year)**	**Material**	**Sample**	**Method**	**Subject research**	**Outcomes**
**Hur et al., 2007**	Mixed essential oils *of rose* ,*lavender, * *rose geranium *	Women between 45 and 54 years of age	Aroma-massage therapy	Blood pressure Lipid metabolism	↓SBP and ↓DBP in the aroma massage therapy group (SBP: p <0.05; DBP: p <0.05). No significant differences in lipid metabolism between two groups HDL (p <0.01) and TG (p< 0.05).
**Fukui et al., 2007**	*R. damascena oil* (0.03 ml*)*	Healthy college students	Aroma therapy	Endocrine system	↓Levels of cortisol in males and females. ↓Testosterone in the female subjects.
**Kim et al., 2011**	Mixed essential oils of *Rosa centifolia,* *Rosa damascena, Salvia sclarea Pelargonium graveolens, Zingiber officinale* (at the concentration of 3%.)	Female nurses	Aroma-massage therapy	Menstrual pain	↓ Menstrual pain (p < 0.001). ↓ Level of anxiety (P = 0.001).
**Farnia et al., 2015**	*R. damascena oil* (contained 17 mg Citronellol)	Male suffering from MDD and SSRI-I SD	Aromatherapy	Sexual dysfunction	↓Sexual dysfunction (p<0.05).
**Farnia et al., 2015**	*R. damascena oil* (contained 17 mg Citronellol)	Female suffering from MDD and SSRI-I SD	Aromatherapy	Sexual dysfunction	↓Sexual dysfunction (p<0.05).
**Ayan et al., 2013**	*R. damascena oil* (maintained at a 2% concentration)	patients with renal colic	Aromatherapy	Pain	↓ Pain intensity 10 and 30 minutes after treatment. (p = 0.002, p = 0.000).
**Marzouk et al., 2013**	Essential oils: *rose, cinnamon, clove, and lavender* (diluted in sweet almond oil at a final concentration of 5%)	Nursing students	Aromatherapy	Menstrual pain	↓The level (p= 0.007) and duration (p= 0.007) of menstrual pain and the amount of menstrual bleeding.
**Sadeghi et al., 2015**	*R. damascena oil* (4% diluted in almond oil)	Female nurses	Aroma-massage therapy	Menstrual pain	↓Pain severity (p = 0.000).
**Haze et al., 2002**	*R. damascena oil*	Healthy females	Aromatherapy	Sympathetic activity	↓30% in adrenaline concentration (P = 0.01) and ↓ 40% in relative sympathetic activity (P= 0.01).
**Igarashi et al., 2014**	*R. damascena oil* (0.2 L) was injected to a 24-L odor bag	Female university students	Aromatherapy	Evaluations of relaxation	↑‘‘comfortable’’, ‘‘relaxed’’ and ‘‘natural’’ feelings ↓The mean oxy-Hb concentration in the right prefrontal cortex (p<0.05).
**Marofi et al., 2015**	*R. damascena oil*	Children hospitalized for surgery	Aromatherapy	Postoperative pain	↓Pain intensity in each time point of 3, 6, 9, and 12 h after arrival to the ward (p < 0.05).
**Kheirkhah et al., 2014**	*R. damascena oil*	Nulliparous women	Aromatherapy	Anxiety	↓Anxiety score in transitional and active phase (p<0.001).
**Hongratanaworakit, 2008**	*R. damascena oil* (1 ml of a 20% (w/w) solution of rose oil in sweet almond oil)	Healthy volunteers	Massage therapy with rose oil	Autonomic parameters and emotional responses	↓SBP, BR, BOS (p<0.03). No significant effects on DBP and on PR (p>0.05). ↓Alertness, ↑calmness, ↑ relaxation (p=0.03 for all). No significant effects on attentiveness, mood and vigor (p>0.05 for all).

BP: Blood Pressure, SBP: Systolic Blood Pressure, DBP: Diastolic Blood Pressure, HDL: high density Lipid, TG: Triglyceride, MDD: Major Depressive Disorder, SSRIs: Selective Serotonin-Reuptake Inhibitors, SSRI-I SD: SSRI-induced sexual dysfunction, VAS: Visual Analogue Scale, MAP: Mean Arterial Pressure, BPM: Beats Per Minute, PR: Pulse Rate, BOS: Blood Oxygen Saturation, BR: Breathing Rate, ST: Skin Temperature

**Other effects**

Igarashi et al. (2014) ▶ showed that olfactory stimulation by rose oil induces physiological and psychological relaxation effects. In this study, the participants were exposed to air impregnated with rose oil for 90 seconds. Control subjects were in the same situation but inhaled only air that was not impregnated with rose oil. The results showed that inhalation of rose oil significantly decreases oxy-hemoglobin concentration and activity in the right prefrontal cortex and increases comfortable feeling conditions.

Haze et al. (2002) ▶ found that inhalation of rose oil decreases relative sympathetic activity as measured by heart rate variability and low frequency amplitude of systolic blood pressure in healthy adult females.

According to the Fukui et al. (2007) ▶ study rose oil inhalation decreased salivary cortisol and testosterone levels in healthy participants. Kheirkhah et al. (2014) ▶ investigated the efficacy of *R. damascena* oil in anxiety in the first stage of labor and showed reduction of anxiety in the active phase. Moreover, Hur et al. (2005) ▶ reported that aromatherapy with rose oil could reduce plasma epinephrine and norepinephrine, but does not have significant effect in mother's anxiety. In animal studies, two phytochemicals including sytrinol and 2-phenylethyl alcohol have been shown to be responsible for anxiolytic activity of rose oil (Burns et al., 2000 ▶; Senol et al., 2013 ▶). 

Hongratanaworakit (2009) ▶ showed that topical application of rose oil significantly decreases blood oxygen saturation, breathing rate, and systolic blood pressure in forty healthy subjects. In this study, olfactory stimulation by inhalation was prevented. 

Hur et al. (2007) ▶ in a study on 58 women showed that aromatherapy massage produces significant differences between pre and post-treatment levels of systolic blood pressure. 

## Conclusion

Different therapeutic properties of rose oil have been investigated in human studies and the most important of them are analgesic and anti-depressant activities. No side effects have been reported from rose oil in investigated human studies. According to Persian Medicine, some other pharmacological activities including anti-inflammatory and anti-hemorrhoidal properties have been attributed to this oil; however, no clinical trial has been focused on these activities yet. So, it is suggested to design clinical studies to evaluate these pharmacological activities. Furthermore, more research with higher populations are recommended to investigate the efficacy and safety of treatment with rose oil.

## Conflict of interest

There is no conflict of interests
